# Asystole in the epilepsy unit

**DOI:** 10.1186/s12883-015-0336-y

**Published:** 2015-05-14

**Authors:** Asaf Honig, Shmuel Chen, Felix Benninger, Rima Bar-Yossef, Roni Eichel, Svetlana Kipervasser, Ilan Blatt, Miri Y. Neufeld, Dana Ekstein

**Affiliations:** Department of Neurology, the Agnes Ginges Center of Neurogenetics, Hadassah-Hebrew University Medical Center, Jerusalem, Israel; Department of Cardiology, Hadassah-Hebrew University Medical Center, Jerusalem, Israel; Department of Neurology, Rabin Medical Center, Petach Tikva, Israel; EEG and Epilepsy Unit, Department of Neurology, Tel-Aviv Sourasky Medical Center, Sackler School of Medicine, Tel-Aviv University, Tel-Aviv, Israel; Department of Neurology, Sheba Medical Center, Tel-Hashomer, Israel

**Keywords:** Asystole, Cardiac, Ictal, Pacemaker

## Abstract

**Background:**

Early identification of cardiac asystole as a reason for syncope is of uttermost significance, as insertion of a cardiac pacemaker can save the patient’s life and prevent severe injury. The aim of this work was to emphasize the subtle and unusual presentations of asystole in patients evaluated in epilepsy units.

**Methods:**

We reviewed the clinical presentation, ECG and EEG data of a series of seven patients who were evaluated in four epilepsy units and were diagnosed with asystole.

**Results:**

Three patients had unusual clinical manifestations of cardiac asystole, resembling epileptic seizures. Three patients had asystole induced by epileptic seizures and in one patient the diagnosis was not clear. All patients except one were implanted with a pacemaker and improved clinically.

**Conclusions:**

Seizure-induced asystole is a rare complication of epilepsy and asystole may clinically mimic epileptic seizures. A high level of suspicion and thorough prolonged cardiac and EEG monitoring are mandatory for reaching the right diagnosis. As the diagnosis is rare and difficult to reach, a flow chart to assist diagnosis is suggested.

## Background

Syncope accounts for 1–3 % of the referrals to emergency departments [1] and is characterized by global cerebral hypoperfusion. The resulting neurological signs and symptoms range from transient loss of consciousness (TLOC) to milder consequences of brain dysfunction that can mimic epileptic seizures [2]. Syncope is classified as reflex (vasovagal), orthostatic hypotensive, and cardiac [1]. The latter can be further divided into two major etiological groups: arrhythmia and structural cardiac diseases. Arrhythmia is an important life threatening event. However, its diagnosis is frequently challenging, because of its abrupt appearance and transiency and complete normalization of the ECG between events. Making the correct diagnosis of arrhythmia-induced syncope can be even more difficult in children and young adults lacking relevant family history, due to its rarity in this population.

Patients with TLOC are often referred to neurologists for evaluation. However, as the heart and the brain are functionally interdependent, diagnostic difficulties arise in the clinical practice [3]. For instance, cardiac arrhythmia may lead to decreased circulation to the brain, which can manifest as syncope, sometimes with myoclonic seizures. On the other hand, as cardiac arrhythmias may also ensue secondarily to epileptic activity, partial epilepsy can induce bradyarrhythmias and thereby cause syncope [3]. Therefore, cardiac arrhythmias should be frequently considered in the differential diagnosis of epileptic seizures in patients with TLOC. Although ictal tachyarrhythmias have been most commonly described in patients with epilepsy, bradyarrhythmias (i.e. severe bradycardia and asystole) were observed and have been associated with impaired sympathetic cardiac innervations [4]. Additionally, both cardiac arrhythmias and respiratory arrest have been implicated in the pathophysiology of sudden unexpected death in epilepsy patients (SUDEP) [5].

This study presents a series of seven patients with recurrent TLOC and evidence of asystole, who were evaluated in epilepsy units, particularly stressing the similarities and differences between cardiac asystole (CA) and ictal asystole (IA).

## Methods

We reviewed the clinical presentation, ECG and EEG data of a series of seven patients who were evaluated by the teams of four leading epilepsy units in Israel and were diagnosed with asystole. Three patients were found to have asystole through evaluation in the outpatient clinics and two additional patients through evaluation in the emergency rooms. Additionally, two more patients were found through a retrospective review of the files of over 1,000 patients who underwent video-EEG monitoring between October 2010 and August 2013 in our institutions to find patients who had asystole during monitoring. The study was approved by Hadassah Medical Center ethics committee. Any identifiable details (such as initials, hospital numbers, age, gender, dates of diagnosis or studies) of the patients were omitted from all sections of this article, including the figures. However, patients 1 and 5, whose data are presented in the figures, gave written consent for their clinical details to be published in medical publications.

## Results

Demographic and clinical data of the patients are shown in Table 1. Two of the patients were male and five were female. Patients 2, 3 and 4 presented to outpatients’ clinics for evaluation of TLOC. Patients 1 and 6 were brought to the emergency department due to very frequent TLOC spells and a first ever generalized convulsive seizure, respectively. Patients 5 and 7 were diagnosed with asystole as an unexpected consequence of monitoring for evaluation of drug-resistant epilepsy (out of more than 1,000 patients monitored in the participating epilepsy centers by video-EEG).

**Table 1:** Patients’ data

Pt. no./age at diagnosis	1/40–54y ^a^	2/19–24y ^a^	3/ >55y ^a^	4/19–24y ^a^	5/ >55y ^a^	6/ >55y ^a^	7/ >55y ^a^
History of other medical issues/medications	Healthy/ none	Healthy/ none	GERD, migraines/ omeprazole	Type 1 DM/ insulin, OXC	Osteoporosis/ alendronate, omeprazole, OXC, TPM, CBZ	HTN, DM, obesity/ ramipril, lercanidipine, metformine, simvastatin	Cognitive & behavioral impairment/ haloperidol, CBZ, TPM
TLOC onset (age)/CA or IA	<18y^a^/CA	19–24y^a^/CA	>55y^a^/CA	19–24y^a^/IA	25–39y^a^/IA	>55y^a^/IA	<18y^a^/probable IA
Clinical manifestations of events	Unconscious fall preceded by disorientation, rapid recovery	General weakness sometimes followed by unconscious fall, with rapid recovery and residual transient left extremities weakness	Abdominal discomfort, dizziness, sometimes followed by general weakness and unconscious fall, rapid recovery	Scenes from the past, thoughts and emotions, in a brief run; after 1y unconscious falls, sometimes preceded by same feelings	Loss of contact, sometimes preceded by general weakness and sometimes followed by unconscious fall, prolonged recovery. In the past – GTCSs.	Unconscious after GTCS followed by right Todd’s palsy, multiple TLOC events with cyanosis, some evolving after right sided convulsions	Loss of contact, oral and right arm automatisms, sometimes followed by bilateral convulsions. Episodes of gait instability sometimes followed by falls.
Frequency of events	Many/day every few months to years	Weakness-once/week, fall-once/2 weeks	Preceding symptoms-once/3–4 weeks, fall-once/ 2 months	Monthly	Falls-twice a week, daily contact loss	Acute on presentation to ER, 1–5/h for several hours	Daily
ECG findings/longest asystole duration/captured by	complete AV block/ 25 s/ VEEG for characterization of events	complete AV block/ 4.5 s/ Holter ECG	complete AV block/7 s/ ECG loop recorder	bradycardia evolving into asystole/ 22 s/ VEEG for characterization of events	bradycardia evolving into asystole/ 15 s/ VEEG for DRE	bradycardia evolving into asystole/ 10 s/ ECG monitor	bradycardia evolving into asystole/56 s/VEEG for DRE
EEG findings	Normal between episodes, generalized slowing and background attenuation during asystole	Normal (awake & sleep deprived)	Normal (awake & sleep deprived)	Left temporal ictal activity, independent left and right temporo-occipital interictal activity	Left temporal ictal and interictal epileptic activity	Left temporal periodic epileptiform discharges	Slow background, right and left fronto-temporal independent interictal activity, right temporal ictal activity
Imaging	Normal (CT)	Normal (MRI)	Normal (MRI)	Normal (MRI)	Left temporal AVM (MRI, angiography)	Left temporal ICH (CT, MRI)	Left MTS, right hippocampal atrophy, white matter microvascular changes, general atrophy (MRI)
Duration of follow-up after pacemaker implantation/outcome	1y/ no TLOC, anxiety developed, off medications	2y/ 1 TLOC during stress, M/P reflex syncope	4 m/ no events	8y/free of all events, on LTG	2y/falls decreased to once/1–2 m, on OXC, TPM, CBZ	1y/ PAF, no seizures or TLOC events, on VPA	No implantation/daily events continue

*TLOC*, transient loss of consciousness; *CA*, cardiac asystole; *IA*, ictal asystole; *GERD*, gastroesophageal reflux disease; *DM*, Diabetes Mellitus; *HTN*, hypertension; *DRE*, drug-resistant epilepsy; *PAF*, paroxysmal atrial fibrillation; *OXC*, oxcarbazepine; *TPM*, topiramate; *CBZ*, clobazam; *LTG*, lamotrigine; *VPA*, valproic acid

^a^to avoid identification of the patients, their exact age was omitted and replaced by age range: less than 18y; 19–24y; 25–39y; 40–54y; more than 55

**Fig. 1:**
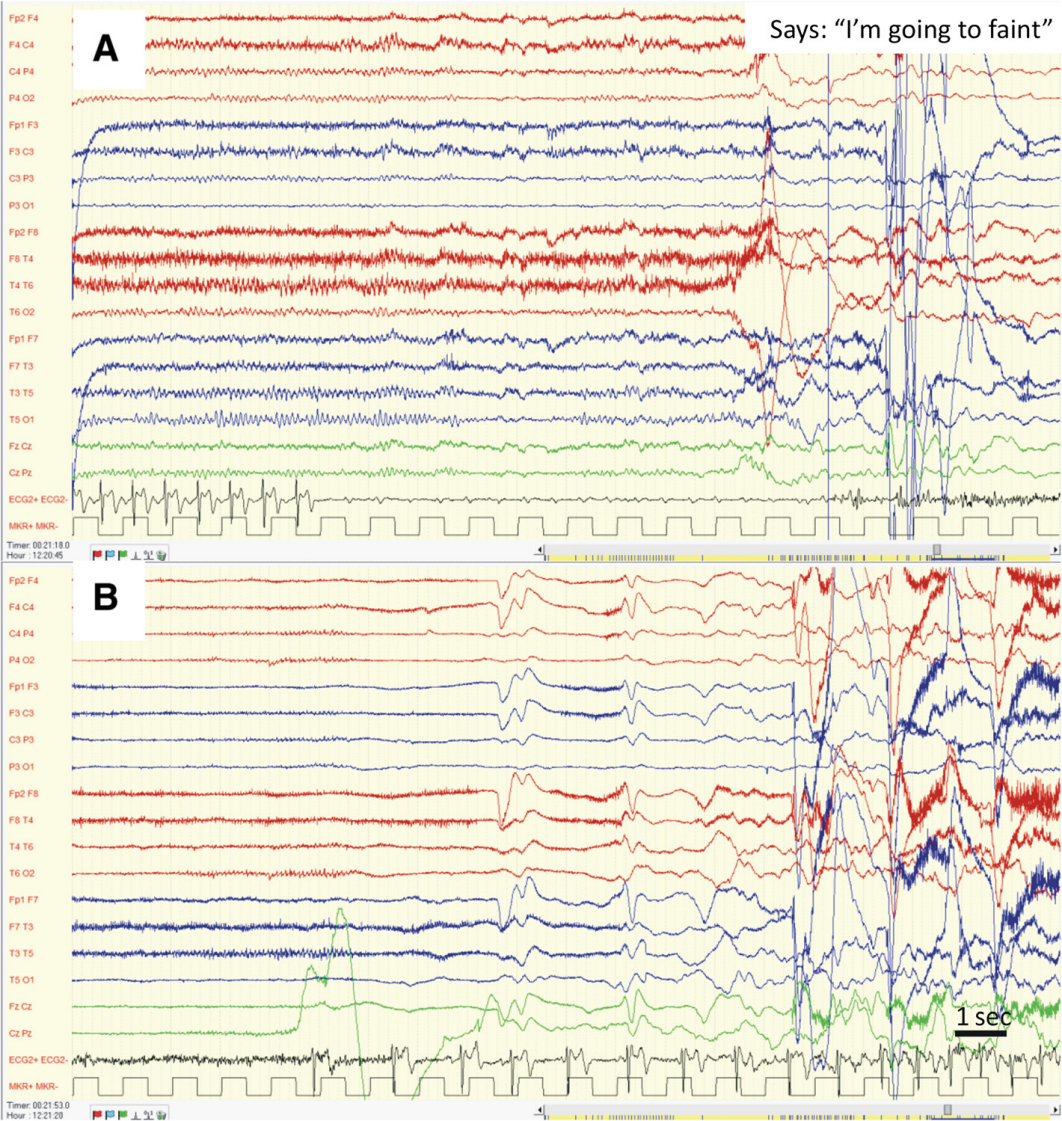
EEG and ECG data during asystole in patients one **a**. Complete AV block emerging out of normal awake EEG. Note the continuation of P waves alone (not followed by QRS complexes) on the ECG. Symptoms appear about 10 s after last QRS complex. **b**. Attenuation of EEG activity during asystole and gradual recovery of EEG activity following return to normal sinus rhythm on ECG

All patients, apart from patient six, whose condition started abruptly, had a clear history of recurrent atonic loss of consciousness and their examination revealed severe bruises and injuries. Patients 1 and 2 had asystole that were falsely diagnosed as epileptic seizures (Fig. 1). They had no family history of arrhythmia or sudden death and their baseline ECG was normal. Their TLOC started at a young age and were accompanied by symptoms and signs attributable to focal neurological deficits (temporal in patient one and frontal in patient two). According to the medical team who witnessed the events of TLOC in patients 1–3 (cardiac asystole) and the video EEG of patient 1 – there were no myoclonic jerks. However, stiffening of the body was clearly witnessed in patients 1 and 2. Moreover, patient two had episodes of unidirectional dystonic posture of the neck mimicking adversive seizure. Patient one had been unnecessarily treated with AED for 35 years, including during her pregnancies. Patient three had TLOC with features attributable to temporal lobe seizures. The asystole were diagnosed by prolonged ECG recordings, without concomitant EEG data. Thus a most probable diagnosis of cardiac and not seizure-induced asystole was made retrospectively, following complete cessation of episodes after pacemaker implantation.

**Fig. 2:**
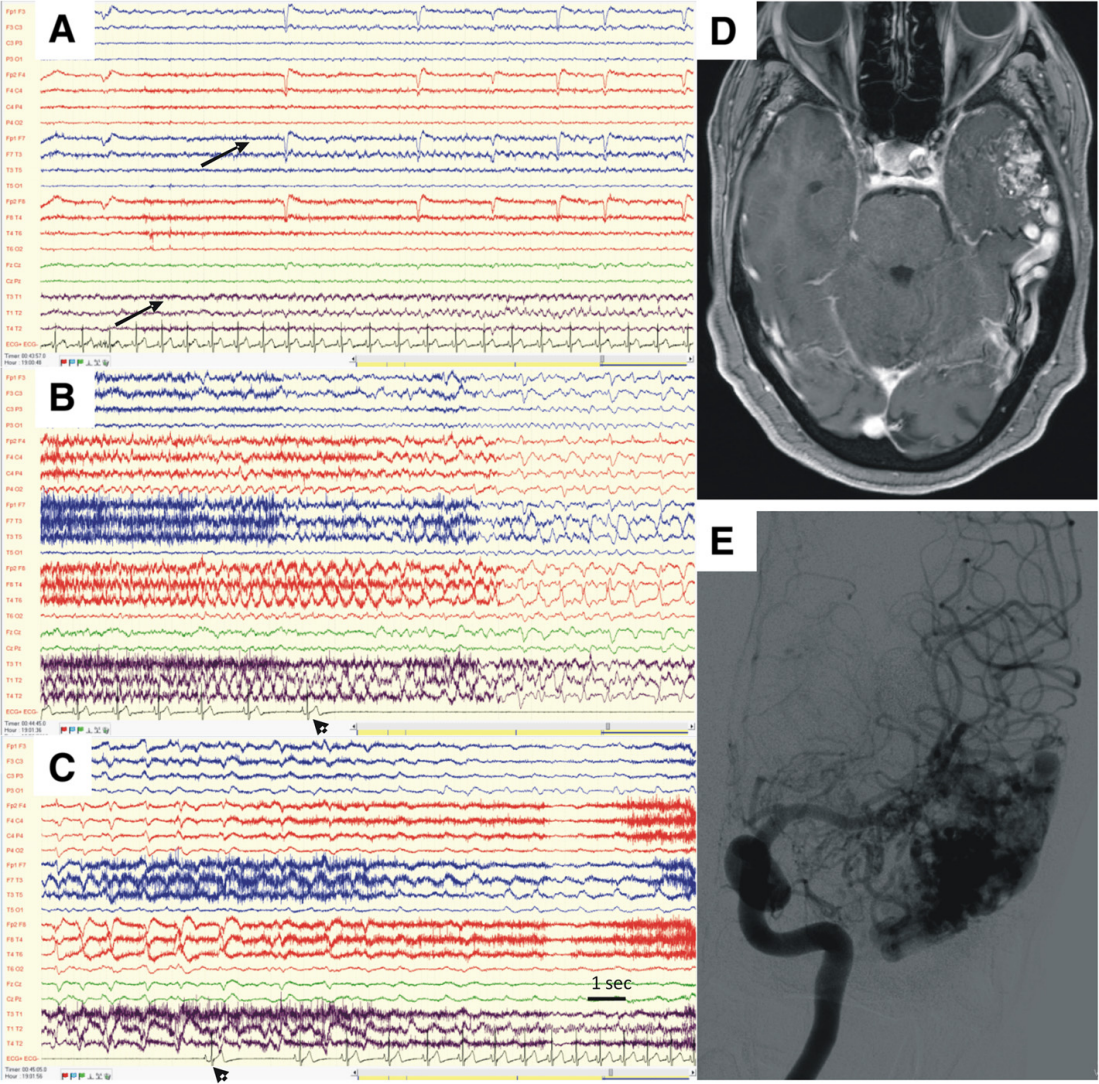
EEG and ECG data during asystole **(a, b, c)**, and brain imaging of patient 5 **(d, e)**. **a**. Onset of left temporal epileptic activity maximally seen on electrodes T1 and F7 (arrows). **b**. Ictal bradycardia evolves into asystole (arrowhead) and EEG changes to a pattern of generalized slowing. **c**. Gradual return of sinus rhythm on ECG (arrowhead) followed by partial recovery of the EEG recording. **d**. MRI of the brain, obtained as T1 protocol with gadolinium, showing a left temporal AVM. **e**. The same AVM is displayed by selective angiography of the left carotid artery

Three patients (4–6) had asystole induced by left temporal lobe seizures (Fig. 2), one of them (patient 6) in the setting of a hemorrhagic stroke and acute symptomatic seizures. This patient also had pre-existing moderately enlarged left atrium and ventricle, and mitral and aortic regurgitation, as well as persistent atrial arrhythmia on follow-up. Patient seven had bilateral fronto-temporal interictal epileptiform activity and left mesial temporal sclerosis, and left temporal semirhythmic EEG activity preceded the asystole. This patient had one additional fit during EEG monitoring, associated with clear right ictal activity. However, during this event the patient felt unsteady and fell, and tachycardia was recorded on ECG.

All patients were implanted with permanent pacemakers, besides patient seven (who refused). The implantation of a pacemaker abolished all previously reported symptoms in patients with cardiac asystole, and decreased falling in a patient with epilepsy (patient 5).

## Discussion

In this series of seven cases we described together patients with both CA and IA, with the common feature of being evaluated by epileptologists. We believe that this enabled us not only to show variable clinical presentations of IA, but also to emphasize a large array of potential pitfalls encountered on the way to diagnosis of challenging cases of TLOC by neurologists. Finally, based on our series and on previously published data from the literature, we propose a flow chart to be used as a diagnostic tool in similar cases.

Most instances of syncope are readily diagnosed, based on clinical characteristics [2]. However, patients 1–3 illustrate the diagnostic difficulties that are sometimes faced in practice. On one hand, the lack of classical prodromal symptoms and their heavily bruised body raised the suspicion for a type of TLOC other than reflex syncope. On the other hand, the young age of patients 1 and 2 at presentation and the neurological symptoms made epilepsy a more plausible diagnosis than CA, even in the absence of epileptic activity on interictal EEG and of abnormalities on brain imaging. This assumption was further supported by the absence of family history of arrhythmias and of personal history of cardiac symptoms. However, the insistence of the treating physician for performing a more thorough investigation in patient 2 prevented commitment to prolonged antiepileptic treatment.

Cardiac arrhythmia was a more likely diagnosis for patient three, given the older age, and it was achieved by prolonged ECG monitoring that captured the clinical events, which were associated with CA. However, since no EEG was concomitantly available, the possibility of IA, given the prodrome and the stepwise increase in onset of epilepsy after the age of 55, could not be excluded on clinical basis alone. Only after the cessation of all types of paroxysmal events after permanent pacemaker implantation, we assumed that the diagnosis of this patient was CA. However, it has been reported [6] that IA might be the only sign of epileptic seizures and amenable to pacing. Therefore, when possible, co-registration of EEG and ECG should be obtained as gold-standard.

Patients 4, 5 and 6 had IA, one (patient 4) while monitored for characterization of events with atonic falls, one (patient 5) during evaluation of drug-resistant epilepsy and one (patient 6) while in the emergency department in the setting of an acute stroke. IA has been well described and has mainly been associated with epileptic foci in the left temporal lobe [7–12]. This localization is consistent with intraoperative electrophysiologic data that attributed parasympathetic effects to stimulation of the left insular cortex [13]. Epilepsy patients who developed IA had different levels of disease control, disease durations, and types of structural pathologies [7–12]. However, though cardiac complications in chronic as well as acute brain conditions have been extensively reported [14], patient six from our series is the first case that shows IA as part of an acute brain insult.

Although a diagnosis of IA was most likely for patient seven, it could not be established due to the lack of clear ictal activity preceding the asystole on scalp EEG. This case demonstrates the limitations that are encountered even when the more sophisticated diagnostic tools are used, mandating for repeated testing in equivocal circumstances.

A high level of suspicion is required to discover IA and indeed most cases in the literature were diagnosed by chance, during video-EEG monitoring, similarly to patients 5 and 7. However, several clinical clues may advocate for an active investigation to capture IA and a series of other characteristics may favor CA as the most likely diagnosis. As both IA and CA are extremely rare complications we propose a practical flow chart for assessment by the neurologist, based on our patients and on other cases from the literature. Three main groups of patients will come to the attention of the neurologist or the epileptologist and each warrants a different series of evaluations and considerations:

1. *Patients with known epilepsy.* Among this group, patients who should be suspected as having IA are those with atonic TLOC events that are not part of their epileptic syndrome. Cases with focal epilepsy, mainly temporal and especially with ictal spread to the left temporal or insular areas [10], have most frequently been associated with development of IA. Furthermore, previous reports and systematic reviews indicate that not the seizure-onset in the left temporal lobe or hemisphere, but rather the seizure-spread to the contralateral hemisphere leads to ictal asystole [5]. Prior cardiac disease causing conduction abnormalities, similarly to case six in our series and to a case with 1^st^ degree AV block [15], or a decreased sympathetic tone [4], such as MI [10, 15] and possibly autonomic diabetic neuropathy (in patient 4 of our series) should be regarded as risk factors for IA. High frequency of events may be best initially evaluated by video-EEG monitoring and low frequency of events-by a 48 h Holter ECG followed by a prolonged cardiac monitoring, as needed. Gradual development of bradycardia culminating in asystole would then suggest IA (as illustrated in our cases and others’ [7, 10]), even in the absence of concomitant epileptic symptomatology, rather than unrelated CA. In order to disclose a culprit structural lesion a good updated imaging is a requisite. As patients with IA also had ictal bradycardia [10], we presume that patients responding to an epileptic attack by bradyarrhythmia instead of tachyarrythmia may bare an increased risk of developing ictal asystole. However, in order to categorically establish such a link further clinical data are needed.
2. *Patients with an acute structural brain lesion (stroke, traumatic brain injury).* Patients with hemorrhagic elements within the lesion and those with cortical involvement are known to be more prone to develop acute epileptic seizures. Case six in our series is the first to exemplify a further complication of such acute symptomatic seizures, namely IA. Similarly, a subacute left subinsular embolic stroke due to LICA dissection was among the first causes of ictal bradycardia described [16]. We suggest that especially when the acute lesion lies in proximity to the left insular cortex the patients should be closely monitored for life threatening arrhythmia. Interestingly, in a study of 655 patients with ischemic stroke, those with left parietal strokes had an increased risk for sudden cardiac death [17].
3. *Patients without epilepsy or a known brain lesion undergoing a primary evaluation of recurrent TLOC*. Here, we stress the significance of achieving a positive etiological diagnosis, especially in the patients with atonic falls and severe bruises. We should not let subjective minor and transient neurological descriptions lead us to a wrong assumption of epilepsy. The diagnostic decision should relay on solid evidence of reliable witnessed seizure, hopefully aided by abnormal EEG or imaging. If this is not the case, prolonged cardiac monitoring is advised and an abruptly appearing asystole would suggest CA rather than IA.

All our patients but one (patient 7) were implanted with pacemakers and the symptoms associates with asystole were abolished. Patient five reported a marked decrease in falls, as previously reported in IA [9]. Pacemaker implantation for IA has been debated and a treatment algorithm was proposed to aid decision making in difficult cases [12]. Some authors [3] argued that cardiac pacing in patients with IA may be reserved for those in whom AED treatment had failed to abolish the TLOC events. In our series, patient 5 had had drug resistant seizures prior to pacing and patient six presented with multiple very frequent asystole. Patient four was treated with cardiac pacing without a period of observation on solely AED treatment.

The main limitation of our study is the small sample size. However, the aim of this work was to describe a diversity of cases, with both cardiac and epileptic etiologies of asystole, indicating various clinical expressions and challenges. The importance of asystole in the neurological practice may warrant combining international efforts to provide more extensive and homogeneous data and build management tools for the rare cases of IA. Additionally, routine EEG monitoring of patients with acute brain injury and in neuro-intensive care units may spread light into the even more rare cases of asystole induced by acute symptomatic seizures.

## Conclusions

In summary, the findings presented in this study emphasize the importance of a high level of suspicion for detecting arrhythmias in general and asystole in particular in selected patients with TLOC, with or without epilepsy. Furthermore, they stress the significant impact of insisting to achieve a positive etiological diagnosis, by performing repeated and prolonged ECG and EEG monitoring, in patients with TLOC.

### Ethical considerations

The study was approved by Hadassah Medical Center ethics committee. Any identifiable details of the patients were omitted from all sections of this article, including the figures. However, patients 1 and 5, whose data are presented in the figures, gave written consent for their clinical details to be published in medical publications.
